# Hospital admissions among adolescents in out-of-home care or special educational needs in England: insights from the ECHILD database of linked health, education and social care records

**DOI:** 10.23889/ijpds.v9i5.2642

**Published:** 2024-09-18

**Authors:** Ruth Blackburn, Ania Zylbersztejn, Dora Kokosi, Michelle Heys, Ruth Gilbert

**Affiliations:** 1 UCL

## Objective

We quantified planned and unplanned hospital admissions during adolescence for children with special educational needs (SEN) or placed into the care of the local authority (child looked after in out-of-home care; CLA).

## Approach

We analysed linked hospital, education and social care records with whole nation coverage of England (ECHILD). Our cohort comprised pupils who started secondary school between 2007/8 and 2011/12 (aged 11), with follow-up until 2018/19 (up to 17-22 years old). Hospital admission rates were examined by sex across distinct groups with varying levels of support: i) no support, ii) school-based SEN only, iii) higher SEN (an Education and Health Care Plan) only, iv) CLA only, v) SEN and CLA, and vi) higher SEN and CLA. Negative binomial regression models were used to estimate rate ratios (RR) for each group relative to no support.

## Results

The study population totalled 2,827,464 children; 65% (1,835,404) had no support, 30% (848,620) SEN only, 3% (93,620) higher SEN only, 1% (30,880) SEN and CLA, with the remaining groups accounting for <1%. Planned and unplanned hospital admission rates were higher for girls than boys. Planned admission rates were highest among younger children in both higher SEN groups. CLA groups had the highest rates of unplanned hospital admissions, especially girls aged 13-15 years with higher SEN (RR: 7.69, 95% CI: 6.86-8.65). A significant proportion of all unplanned admissions in adolescence were mental health-related.

## Implications

Our findings highlight significant unmet health needs, particularly in mental health, among care-experienced adolescent girls with SEN in their mid-teens.

